# Is the racial composition of your surroundings associated with your levels of social dominance orientation?

**DOI:** 10.1371/journal.pone.0186612

**Published:** 2017-10-19

**Authors:** Helena R. M. Radke, Matthew J. Hornsey, Chris G. Sibley, Michael Thai, Fiona Kate Barlow

**Affiliations:** 1 Institute for Psychology, University of Osnabrück, Osnabrück, Germany; 2 School of Psychology, University of Queensland, Brisbane, Queensland, Australia; 3 School of Psychology, University of Auckland, Auckland, New Zealand; 4 School of Applied Psychology, Griffith University, Mt Gravatt, Queensland, Australia; Institut Català de Paleoecologia Humana i Evolució Social (IPHES), SPAIN

## Abstract

We investigate the extent to which minority group members are surrounded by outgroup members in their immediate environment as a predictor of social dominance orientation. Using a large representative sample of New Zealanders, we found that minority group members in outgroup dense environments reported lower levels of social dominance orientation (Study 1). In studies 2 and 3, Asian Australian and Black American participants who were surrounded by outgroup members reported lower social dominance orientation. For majority group (White) participants there was no association between social dominance orientation and outgroup density. Study 4 explained the overall pattern: Black Americans surrounded by outgroup members perceived their group to be of lower status in their immediate environment, and through this, reported lower social dominance orientation. This article adds to growing literature on contextual factors that predict social dominance orientation, especially among minority group members.

## Introduction

Few studies have investigated social dominance orientation (SDO) in minority groups. This is surprising given that a portion of disadvantaged minority group members support hierarchical social structures that ostensibly marginalize them [1,2]. In the current paper, we make the case that the extent to which minority group members are surrounded by outgroup members is a (previously unexplored) predictor of SDO. We propose that minority group members who are *not* surrounded by fellow ingroup members will see their own group as comparatively disadvantaged in their immediate environment, and this will be associated with a decrease in the extent to which they support hierarchical social structures.

### Social dominance orientation

SDO is an attitudinal outlook whereby a person orients towards propping up hierarchical social structures and opposing group-based equality [3]. Theoretically, SDO is a strategy by which people can prevent intergroup conflict by propagating legitimizing myths that justify the dominance of some groups over others. Consistent with this, people high in SDO are more likely to endorse politically conservative ideologies, hold prejudicial attitudes towards lower-status groups, and endorse hierarchy-enhancing practices and policies such as capital punishment [3–5]. For people in positions of power, or for members of high status groups, a socially dominant orientation is a way to maintain their influence and authority. SDO among someone who belongs to a group that is marginalized by the hierarchy (e.g., Asian Australians, Black Americans) has received less scholarly attention.

One exception is the study of SDO in women–most studies that look at the association between SDO and other attitudes include women [3,4], and SDO among women is positively correlated with greater levels of sexism [6,7]. Women, like disadvantaged minority groups, are negatively affected by the social hierarchy [4–7]. There are, however, core differences between the category of *women* (who exist under a patriarchal hierarchy) and other disadvantaged groups that are not based on gender (or age; called *arbitrary-set groups* that exist in an *arbitrary-set hierarchy*; [8]). An example of an arbitrary-set group is those group memberships that are based on a person’s race or religion. An arbitrary-set hierarchy is one that is socially constructed and therefore contingent on the social situation. While complete discussion of these differences is beyond the scope of this paper, Sidanius and Veniegas [8] comprehensively deals with the theoretical and empirical distinctions between these two sets of groups. For SDO in arbitrary-set groups (in this case, members of racial minority groups) very little research exists (although see [9] for an example).

### Social dominance orientation among disadvantaged minority group members

A number of longitudinal studies provide directional evidence for the role of SDO in attitude change [10,11]. In general, SDO scores have been theorized to be higher among dominant group members compared to subordinate group members, given that it is an ideology that serves to justify and protect the status quo [4]. Research has shown that SDO scores can also be readily manipulated by the immediate (rather than chronic) social context [12–15]. For example, members of dominant groups report higher levels of SDO when their dominant status is made salient than when it is not [16]. Similarly, other research has found that SDO levels are subject to change over time, becoming higher for students enrolled in hierarchy-enhancing courses (e.g., business) compared to hierarchy-attenuating courses (e.g., psychology; [17]; see also [18]). Over four studies, Guimond and colleagues [19]) tested the hypothesis that contextual factors, such as a person’s position in the social hierarchy, drives their endorsement of SDO. They found support for this conceptualization of SDO, which in turn supports our argument that 1) SDO is malleable, and 2) one’s position in the immediate social system is a robust predictor of SDO.

Objectively, minority group members such as Black Americans exist in a social system in which they are disadvantaged by the hierarchy (e.g., in terms of wealth, education, and status). As such, it might be expected that SDO is chronically low in minority groups. However, this is not necessarily the case. Sidanius and colleagues [20] for example, argue that Black Americans with a “high level of SDO would not desire Black Americans to dominate White Americans, but rather would desire to maintain the extant hierarchical domination of Blacks by Whites, even at the ingroup’s expense”, and refer to recent empirical evidence supporting this claim [1]. We extend this argument to propose that despite the *objective* reality of the hierarchy, the environments in which minority group members are situated change the *subjective* experience of the hierarchy.

Specifically, we examine outgroup density–the extent to which minority group members are surrounded by outgroup members–as a potential factor associated with SDO among minority group members. In particular, we suggest that for minority group members, being surrounded by fellow minority group members might be protective when it comes to status evaluations of their own group. Conversely, minority group members who are isolated from other ingroup members may find themselves in environments that make salient their relatively low power and status compared to other groups (e.g., the majority group, or other minority groups). From a social identity perspective [21], outgroup density can be described as a group-level *other-total* ratio (where the number of people in the outgroup is divided by the number of people in the outgroup and the ingroup combined). Past research has found that as an individual’s group becomes larger (and the other-total ratio decreases), they experience deindividuation. However, as an individual’s group becomes smaller (and the other-total ratio increases) the ingroup becomes more salient to the individual. We would expect the embedded and immediate reminders of a social hierarchy *not* in a minority group’s favor would lead to relatively low endorsement of socially dominant ideals, and comparatively high endorsement of equality ideals. In other words, when the other-total ratio is small, the minority group (and its disadvantage) becomes salient. Some research tangentially supports this idea; for example, Black Americans endorse hierarchical social relationships when they perceive the social system to be fair [2], and Black Americans who live in minority dense neighborhoods report less perceived discrimination [22].

This paper has three cases for theoretical impact. First, it adds to a small but growing literature examining the contextual factors that are associated with SDO. Second, it does so by examining a structural factor–outgroup density–that has received little attention in social psychology. Finally, because the overwhelming focus of SDO research has been on majority group members, we help fill an empirical vacuum regarding variation in SDO among minority group members. To ensure that any effects of outgroup density were not due to covariation with demographic variables we conducted analyses with participant age (all studies), gender (all studies), level of education (studies 1, 3, and 4), and socio-economic status (studies 3 and 4) entered as controls.

Across the first three studies we aimed to test whether outgroup density was negatively associated with SDO among Māori, Pacific peoples, and Asian peoples in New Zealand (Study 1), Asian Australians (Study 2), and Black Americans (Study 3). For each study we also tested whether outgroup density negatively predicted SDO among majority (i.e., White) group members. The aim of Study 4 was to show that a decrease in perceived status explained the negative relationship between outgroup density and SDO among Black Americans. The data confirmed our expectations across the four studies.

## Study 1

Study 1 employed national data to establish the association between the objective outgroup density of the different areas of New Zealand (based on census data about regions) and the levels of SDO of a large-scale national probability sample of minority participants living in those regions. Data were drawn from the 2009 (Time 1) wave of the New Zealand Attitudes and Values Study (NZAVS).

## Method

### Participants

Participants were 1217 nationally representative ethnic minority (non-European) group members sampled as part of the NZAVS. Participants were 779 Māori (the indigenous peoples of New Zealand), 141 Pacific peoples, and 246 Asian peoples, all living in New Zealand. Fifty-one participants identified as a combination of Māori, Pacific peoples and/or Asian peoples and were asked to indicate their ethnic group affiliation in terms of priority. Participants were 737 women and 480 men, and had a mean age of 42.89 years (*SD* = 13.84).

### Sampling procedure

The Time 1 (2009) NZAVS contained responses from 6518 participants sampled from the 2009 New Zealand electoral roll. The electoral roll is publicly available for scientific research and in 2009 contained 2,986,546 registered voters. This represented all citizens over 18 years of age who were eligible to vote regardless of whether they chose to vote, barring people who had their contact details removed due to specific case-by-case concerns about privacy. The sample frame was spilt into three parts. Sample Frame 1 constituted a random sample of 25,000 people from the electoral roll (4060 respondents). Sample Frame 2 constituted a second random sample of a further 10,000 people from the electoral roll (1609 respondents). Sample Frame 3 constituted a booster sample of 5500 people randomly selected from area units of the country with a high proportion of Māori, Pacific Nations and Asian peoples (671 respondents). A further 178 people responded but did not provide contact details and so could not be matched to a sample frame.

In sum, postal questionnaires were sent to 40,500 registered voters or roughly 1.36% of all registered voters in New Zealand. The overall response rate (adjusting for the address accuracy of the electoral roll and including anonymous responses) was 16.60%.

### Area-unit information

New Zealand is unusual in that it has rich census information about each area unit/neighborhood of the country available for research purposes. The NZAVS matches each participant to their area unit, and hence it is possible to look at the associations between characteristics of areas and the participants who live in the area. Following the recommendations outlined by Sibley [23], we focused on information at the area unit level. New Zealand is divided (for statistical purposes) into 2020 small geographic area units, each with a mean population of 2210 people (*SD* = 1673, median = 1977). These units are built up from smaller ‘meshblocks’, which abut one another and are defined geographic areas that are intended to be approximately equal in population density. Hence, the area units are geographically smaller in urban areas, and larger in rural areas where population density is lower (see Sibley [23] for technical details about the use of geographic information in the NZAVS).

The New Zealand Census provides information about the number of people who identify with each broad ethnic group (European, Māori, Pacific, Asian) in each area unit. We used the information from the 2006 Census to calculate the proportion of outgroup members in each of the 2020 area units in New Zealand for each minority group (i.e., Māori, Pacific, Asian). Our analysis included a total of 744 area units. This was less than the total available units because our analysis was limited to ethnic minority group participants. Ethnic minority groups are not evenly distributed across New Zealand, and hence only areas in which we sampled ethnic minority group participants were included in the analysis. The mean proportion of outgroup members in the area units assessed for which participant data were available was .79 (79% outgroup; *SD* = .18, range = .05 –.99). For participants who identified as a combination of Māori, Pacific peoples, and Asian peoples, we used the group they prioritized as their ethnic group affiliation to create a measure of outgroup density.

### Questionnaire measures

In all studies responses were made on a 7-point scale ranging from 1 (*strongly disagree*) to 7 (*strongly agree*) unless otherwise indicated. In Study 1, SDO was measured using six balanced items from the SDO6 scale (e.g., “It is OK if some groups have more of a chance in life than others”, “Inferior groups should stay in their place”, “To get ahead in life, it is sometimes okay to step on other groups”; *M* = 2.57, *SD* = .95, α = .69; [4]). Mean levels of SDO were 2.72 when outgroup density was between 0 and 49.90%, and 2.56 when outgroup density was between 50% and 100%. Due to time constraint, it was not possible to include the full measure of SDO in studies 1, 2, and 3. We also measured level of education from 0 (*no qualification*) to 10 (*doctorate degree*). See Sibley, Greaves, and Milojev [24] for information about survey items.

### Ethics statement

The study was approved by the University of Auckland Human Participants Ethics Committee. Prior to participating in the study participants were given an information sheet detailing the purpose of the study, what was involved in participation, how long the data would be stored, and how the data would be used. Participants then provided signed consent. No data was retained or analyzed without signed consent. The data from Study 1 cannot be made available due to ethical restrictions imposed by the University of Auckland Human Participants Ethics Committee. A de-identified dataset is available upon request from the University of Auckland for all appropriately qualified researchers. More information regarding requests for data access may be found here: https://www.auckland.ac.nz/en/about/research/re-ethics/re-uahpec.html

## Results and discussion

We conducted a regression assessing the link between the proportion of outgroup members in area units based on census data and the level of SDO of minority group members living in those neighborhoods. As shown in Table 1, minority group members who lived in regions with a higher overall proportion of outgroup members tended to have lower levels of SDO when adjusting for participants’ gender, age, and level of education.

**Table 1:** Regression assessing the link between outgroup density and the level of SDO of minority group members (Study 1).

	*b*	*se*	*t*
Intercept	2.61	.16	16.34***
Outgroup density (0–1)	-.32*	.16	-2.03*
Level of education (0–10	-.00	.01	-.21
Gender (1 male, 0 female)	.29	.06	5.12***
Age (years)	.00	.00	1.18

*N* = 1217 participants in 744 area units.

*p < .05,

***p < .001.

*t* = t-value representing the difference from the null hypothesis.

We also tested whether outgroup density predicted SDO for White participants surveyed at Time 1 (2009) of the NZAVS (*N* = 3797; 61% female; *M*_*age*_ = 48.47; *M*_*SDO*_ = 2.56; *SD*_*SDO*_ = .97). All measures were as per those reported for the minority group sample reported above. We found that outgroup density did not predict SDO for White New Zealanders when controlling for participant age, gender, and level of education (*b* = .06, *b*_*SE*_ = .13, *t* = .44, *p* = .659).

In Study 1, then, we establish our core proposed effect. Minority group members who live in objective environments (as indexed based on New Zealand census data) where there are a high proportion of outgroup members also reported lower levels of SDO. Thus, Study 1 provides the first test of the link between outgroup density and minority group members’ level of SDO.

## Study 2

In Study 1 we established the core effect. In Study 2 we wished to extend our findings, drawing on data from a different national and ethnic minority group. Specifically, we used an Asian Australian sample to investigate the relationship between outgroup density and SDO.

## Method

### Participants

A total of 292 Asian Australian students (56% female; *M*_age_ = 21.03) were recruited via mass emails and snowball sampling from social networking websites (e.g., *Facebook*) as part of a large study of Asian Australians. Work using a subset of this larger dataset has previously been published [25]. Our article does not constitute dual publication because our research question is different, we used separate measures from those reported in the previously published paper, the data analysis conducted was different, and as a result, our conclusions are unrelated to those reported in the previously published paper.

### Measures

#### Outgroup density

Outgroup density was measured using two items asking participants to estimate what percentage of their neighborhood and educational institution consisted of other Asians (*r* = .17, *p* = .004). A measure of outgroup density was then calculated by subtracting this value from 100%. Outgroup density was analyzed in decimal form (i.e., from 0–1).

To account for the small correlation between the outgroup density items, we separately tested the correlation between the two items and SDO. The relationship remained significant when using either item as the predictor. It should also be noted that the correlation between the outgroup density items is substantially larger in Study 3 and 4 as compared to Study 2.

#### SDO

SDO was measured using six items from Pratto and colleagues ([3]; e.g., “To get ahead in life, it is sometimes necessary to step on other groups”, “Superior groups should dominate inferior groups”, “Some groups of people are just more worthy than others”; α = .82). Mean levels of SDO were 2.90 when outgroup density was between 0 and 49.90%, and 2.44 when outgroup density was between 50% and 100%.

#### Ethics statement

The study was approved by the University of Queensland Human Participants Ethics Committee. Prior to participating in the study participants were given an information sheet detailing the purpose of the study, what was involved in participation, how long data would be stored for, and how the data would be used. Informed consent was indicated by continuation of the study beyond the information page. The University of Queensland Human Participants Ethics Committee approved informed consent being obtained in this way. The studies were advertised for Asian Australians and White Australians. We complied with the terms of service for the websites from which this data was collected.

## Results and discussion

As can be seen from the correlations summarized in Table 2, outgroup density was associated with lower SDO (*r* = -.19, *p* = .001). This relationship remained when controlling for participants’ age and gender (β = -.18, *p* = .002). As such, the results of Study 2 support our core assertion: that outgroup density should negatively predict SDO for minority group members (see S1 Dataset).

**Table 2:** Means, standard deviations, and zero-order intercorrelations for the Asian Australian sample (Study 2).

	*Mean (SD)*	1	2	3
1. Age	21.03 (4.36)	-		
2. Gender	0.57 (0.50)	-.00	-	
3. Outgroup density	0.71 (0.15)	-.03	.06	-
4. SDO	2.49 (1.06)	.02	-.23***	-.19**

**p ˂ .01,

***p < .001.

Male = 0, Female = 1.

We also tested whether the observed patterns held for a sample of White Australian students (*N* = 264; 78% female; *M*_age_ = 21.73). Participants were asked to estimate what percentage of their neighborhood and educational institution consisted of other White Australians (*r* = .24, *p <* .001). Outgroup density was then calculated as described above. The SDO items formed a reliable scale (α = .79). Being surrounded by outgroup members was not associated with SDO for White Australians (*r* = -.09, *p* = .142; see Table 3). This relationship remained non-significant when controlling for participants’ age and gender (β = -.09, *p* = .166; see S2 Dataset).

**Table 3:** Means, standard deviations, and zero-order intercorrelations for the White Australian sample (Study 2).

	*Mean (SD)*	1	2	3
1. Age	21.73 (6.60)	-		
2. Gender	0.78 (0.42)	-.03	-	
3. Outgroup density	0.36 (0.15)	-.09	.15*	-
4. SDO	2.12 (0.88)	-.15*	-.14*	-.09

*p < .05.

Male = 0, Female = 1.

## Study 3

In Study 3 we replicated Study 2 by examining SDO among minority group members with a sample of Black Americans. Again, we hypothesized that outgroup density would be linked to decreased SDO. In addition to controlling for participants’ age and gender, we improve on our controls from Study 2 by controlling for socio-economic status and level of education.

## Method

### Participants

One hundred and two Black Americans (53% female; *M*_age_ = 28.00) were recruited via an online scientific survey pool (www.socialsci.com). The majority of participants reported that they had an average (25%) or just below average (28%) socio-economic status, and that they had attended some college (31%) or had been awarded a bachelor’s degree (28%). This data was collected as part of a larger experiment containing an unrelated manipulation. Additional data analysis revealed that the results did not differ when controlling for the manipulation.

### Measures

In addition to age and gender, participants’ socio-economic status (1 = *extremely poor* to 7 = *extremely wealthy*) and highest level of education (1 = *less than high school graduate* to 8 = *doctorate degree*) were measured.

#### Outgroup density

Outgroup density was measured as per Study 2 (but adjusting for the fact that this was a non-student sample), using two items asking participants what percentage of their neighborhood and workplace consisted of other Black Americans (*r* = .55, *p <* .001).

#### SDO

SDO was measured using five items adapted from Pratto and colleagues ([3]; α = .79). Mean levels of SDO were 2.92 when outgroup density was between 0 and 49.90%, and 2.27 when outgroup density was between 50% and 100%.

### Ethics statement

The study was approved by the University of Queensland Human Participants Ethics Committee. Prior to participating in the study participants were given an information sheet detailing the purpose of the study, what was involved in participation, how long data would be stored for, and how the data would be used. Signed consent was unable to be obtained for this study because it was conducted online. Instead informed consent was obtained by asking participants to view a webpage that outlined the conditions for taking part in the study and to cross a box if they agreed to the conditions. This process of obtaining informed consent was approved by the University of Queensland Human Participants Ethics Committee. To obtain the Black American and White American samples the study was advertised as a questionnaire for these populations. We complied with the terms of service for the websites from which this data was collected.

## Results and discussion

As can be seen in Table 4, Black Americans who reported living and working surrounded by a high proportion of outgroup members reported lower SDO (*r* = -.28, *p* = .005). This relationship remained when controlling for participants’ age, gender, socio-economic status, and level of education (β = -.28, *p* = .005; see S3 Dataset).

**Table 4:** Means, standard deviations, and zero-order intercorrelations for the Black American sample (Study 3).

	*Mean (SD)*	1	2	3	4	5
1. Age	28.00 (8.26)	-				
2. Gender	0.53 (0.50)	.09	-			
3. SES	3.67 (1.34)	-.04	.14	-		
4. Education	4.07 (1.44)	.37***	-.01	.22*	-	
5. Outgroup density	0.63 (0.27)	-.17	-.08	.01	.06	-
6. SDO	2.45 (1.22)	-.02	.10	.26**	-.02	-.28**

*p < .05,

**p < .01,

***p < .001.

Male = 0, Female = 1. SES = socio-economic status.

We again tested whether the observed patterns held for a sample of White Americans (*N* = 133; 56% female; *M*_age_ = 29.82). The same measures were administered to participants as in Study 2 except participants were asked to estimate what percentage of their neighborhood and workplace consisted of other White people (*r* = .09, *p* = .321). Contrary to Study 2, these two measures were not correlated with one another. We therefore created two separate measures of outgroup density (one measuring the extent to which participants were surrounded by outgroup members in their neighborhood, and the other measuring the extent to which participants were surrounded by outgroup members in their workplace). The SDO items again formed a reliable scale (α = .86). Being surrounded by outgroup members was not associated with SDO for White Americans in both the neighborhood where they lived (*r* = .03, *p* = .711) and their workplace (*r* = -.16, *p* = .073; see Table 5). These findings remained non-significant when controlling for participants’ age, gender, social economic status, and level of education (neighborhood: β = .13, *p* = .150; workplace: β = -.11, *p* = .202; see S4 Dataset).

**Table 5:** Means, standard deviations, and zero-order intercorrelations for the White American sample (Study 3).

	*Mean (SD)*	1	2	3	4	5	6	7
1. Age	29.82 (10.17)	-						
2. Gender	0.57 (0.50)	-.02	-					
3. SES	4.05 (1.20)	.16	-.20*	-				
4. Education	4.61 (1.50)	.24**	-.02	.17*	-			
5. Outgroup density neighbourhood	.30 (.24)	.05	.16	-.28**	-.08	-		
6. Outgroup density workplace	.31 (.29)	.27**	.11	-.03	-.00	.09	-	
7. SDO	2.30 (1.18)	.00	-.15	.13	.10	.03	-.16	-

*p < .05,

**p < .01.

Male = 0, Female = 1. SES = socio-economic status.

## Study 4

Although the previous studies support our core prediction, what remains unknown is *why* minority group members in outgroup dense environments might oppose the stratification of groups within society and support group-based equality. Previous research has found that SDO scores can shift depending on whether the ingroup status is made relevant in a given context [13]. We therefore argue that minority group members who are isolated from other ingroup members may not be protected from chronic reminders of systematic discrimination and low status relative to those minority group members who are surrounded by other ingroup members. In Study 4 we test the full proposed model, in which outgroup density is associated with Black Americans’ perceptions of lower group status in their surrounding environment, which in turn predicts decreased SDO.

## Method

### Participants

A total of 144 Black American (44% female; *M*_age_ = 30.90) were recruited through an online scientific survey pool (www.mturk.com). The majority of participants reported that they had an average (35%) or just below average (26%) socio-economic status, and that they had attended some college (33%) or had been awarded a bachelor’s degree (33%).

### Measures

Outgroup density (*r* = .46, *p <* .001), age, gender, socio-economic status and level of education were measured as per Study 3. In Study 4 the full 16 item measure of SDO [3] was included (α = .94). Mean levels of SDO were 2.73 when outgroup density was between 0 and 49.90%, and 1.99 when outgroup density was between 50% and 100%.

#### Perceived status in the surrounding environment

The proposed mediating variable was measured using four items: Participants rated the extent to which “In my neighborhood and place of work, Black Americans are doing better than other racial/ethnic groups”, “Other racial/ethnic groups are struggling more than Black Americans in my neighborhood and place of work”, “Black Americans are doing alright compared to other racial/ethnic groups in my neighborhood and place of work”, and “I believe that Black Americans are better than other racial/ethnic groups in my neighborhood and place of work” (α = .64).

### Ethics statement

The study was approved by the University of Queensland Human Participants Ethics Committee. Prior to participating in the study participants were given an information sheet detailing the purpose of the study, what was involved in participation, how long the data would be stored for, and how the data would be used. Signed consent was unable to be obtained for this study because it was conducted online. Instead informed consent was obtained by asking participants to view a webpage that outlined the conditions for taking part in the study and to cross a box if they agreed to the conditions. This process of obtaining informed consent was approved by The University of Queensland Human Participants Ethics Committee. We complied with the terms of service for the websites from which this data was collected.

To obtain the Black American sample the study was advertised as a questionnaire for this population. To ensure that the data analyzed contained only Black Americans, we asked participants to indicate their race/ethnicity at the end of the survey. Before answering this question, we told participants that while it was a condition of participation in the survey to be a Black American, it was more important for us to have the correct data. We then asked participants to honestly indicate their race/ethnicity and stated that we would still fully reimburse participants who completed the survey but did not identify as a Black American. All participants who completed the survey were paid and those participants who indicated that they were not a Black American (*n* = 4) were deleted from the data analysis. Moreover, the results from Study 4 are consistent with the previous studies which found a relationship between outgroup density and SDO among minority group members but not majority group members. This provides further evidence that we successfully recruited a sample of Black Americans in this study.

## Results and discussion

**Fig 1:**
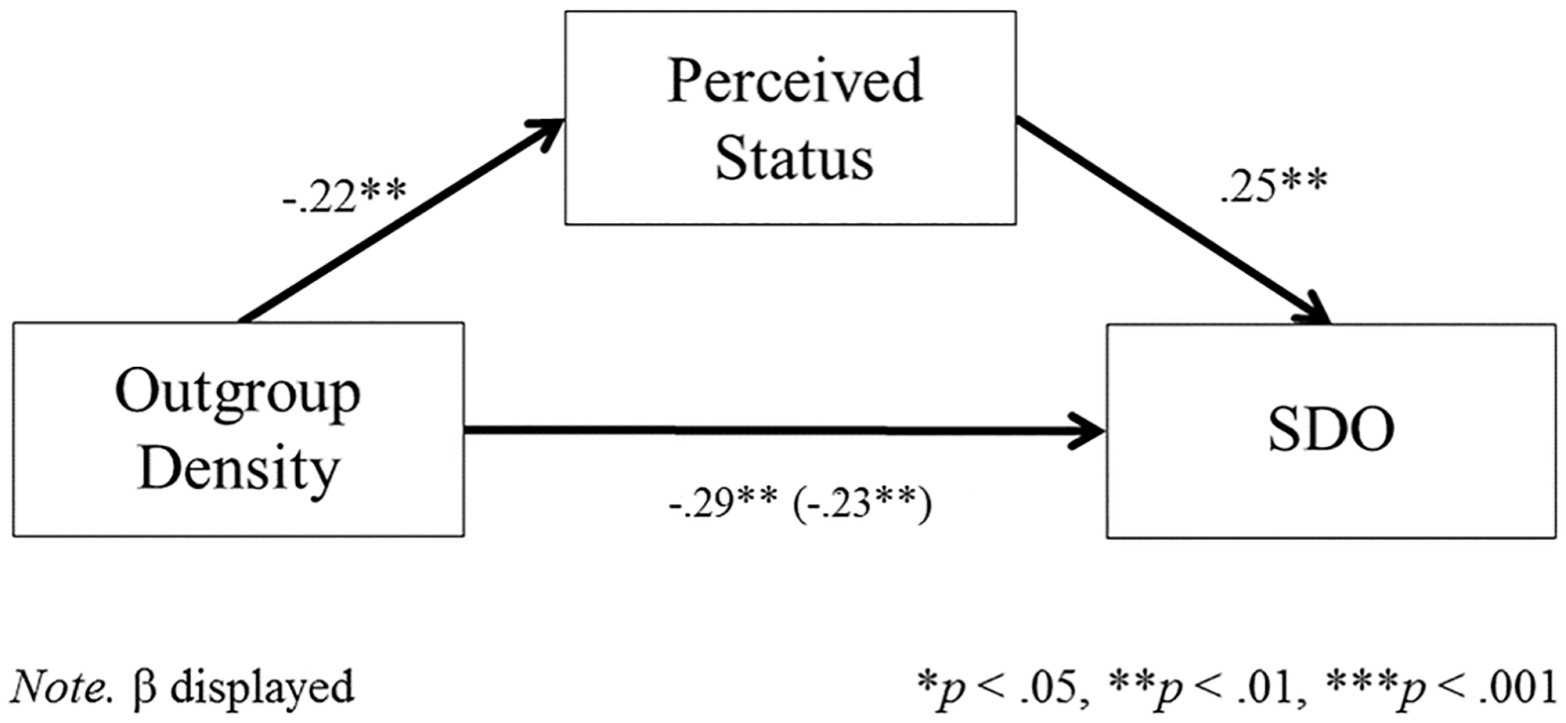
The relationship between outgroup density and SDO mediated by perceived status for the Black American sample (Study 4).

Consistent with the previous studies, Black Americans who reported living and working surrounded by outgroup members reported lower SDO (*r* = -.29, *p* = .001). Critically, Black Americans in outgroup dense environments also reported lower perceived status in their neighborhood and workplace (*r* = -.22, *p* = .007; see Table 6). As can be seen in Fig 1, perceived status mediated the association between outgroup density and SDO. Mediation analyses were conducted using bootstrapping procedures ([26]; SPSS PROCESS macro). We used 5000 bootstrap samples to estimate bias-corrected standard errors and 95% confidence intervals. The indirect effect of outgroup density via perceived status was significant for SDO (*B* = -.24, *B*_*SE*_ = .11; 95% *CI* = -.51, -.07; see S5 Dataset). These results held when controlling for participant age, gender, socio-economic status, and level of education.

**Table 6:** Means, standard deviations, and zero-order intercorrelations for the Black American sample (Study 4).

	*Mean (SD)*	1	2	3	4	5	6
1. Age	30.90 (9.20)	-					
2. Gender	0.44 (0.50)	.11	-				
3. SES	3.68 (1.13)	-.09	-.22**	-			
4. Education	3.88 (1.29)	.15	-.17*	.34***	-		
5. Outgroup density	0.63 (0.24)	-.04	-.03	.03	.05	-	
6. Perceived Status	3.67 (1.05)	-.08	-.07	.15	.04	-.22**	-
7. SDO	2.19 (1.05)	-.11	-.08	.08	.04	-.29**	.30***

*p < .05,

**p < .01,

***p < .001.

Male = 0, Female = 1. SES = socio-economic status.

Study 4 ties the results of the first three studies together, confirming our proposed process. Black Americans who are isolated from other ingroup members perceive their group as more subordinate, and through this, reported lower SDO.

Recent research suggests that SDO might consist of two dimensions; 1) a preference for dominance (SDO-D scale), and 2) a preference for egalitarian intergroup relations (SDO-E scale; [1,27]). We tested these two constructs separately in the mediation model, and found that the results do not change. Outgroup density negatively predicts both the SDO-D and SDO-E scales through lower perceived status of Black Americans.

## General discussion

Over four studies we tested the hypothesis that minority group members in outgroup dense environments are exposed to chronic reminders of their lower status, which in turn is associated with decreased levels of SDO. In line with this prediction, Study 1 found that minority group members who lived in environments that objectively had a high proportion of outgroup members reported lower levels of SDO. Moreover, minority group members (but not majority group members) who were surrounded by outgroup members reported lower SDO in Studies 2 and 3. In Study 4, Black Americans in outgroup dense environments perceived their group as having lower status compared to those in ingroup dense areas. Those who perceived their group as having lower status, in turn, were less likely to support hierarchical social structures in general (SDO). We also found that being surrounded by a high proportion of outgroup members was not related to SDO scores among majority (i.e., White) group members.

Given the emphasis on intergroup relations in social psychology, we seek to extend the psychologically literature by further engaging with the experiences of both advantaged majority and disadvantaged minority group members. Our article contributes to the small amount of literature examining SDO among disadvantaged arbitrary-set groups ([8,9]). In doing so it supports (and extends) past research showing that minority group members’ SDO is dependent on the social context [10,13–16]. While it is commonplace to examine structural and macro-level precipitants of human behavior in sociology, this type of analysis has not been as commonplace among social psychologists.

One exception can be found in the work by Onaret, van Hiel, and Cornelis [28]. These researchers conducted a cross-nation study in which they looked at the association between country-level indices of threat (i.e., gross national product, inflation, unemployment, homicide rate, life expectancy) and indices of right-wing ideology (such as respect for authority and attitudes towards competition). They found that the level of threat in a nation was a robust positive predictor of right-wing attitudes. Extending from this work, we might expect to see high levels of SDO in minority group members who have recently migrated from high-threat/right-wing nations to Europe, for example. In the present studies, the focus was on Māori, Pacific Nations and Asian New Zealanders, Asian Australians, and Black Americans, none of whom fall into that category. Despite this, we have no theoretical reason to expect that the patterns unearthed in this work would be any different if we had investigated different social groups that may fall into this category (e.g., recent migrants from the Middle East or Africa), or groups within different nations. Recent migrants, for example, might be particularly aware of their lower-status compared to outgroup members as they transition to becoming a member of their new society. Nonetheless, a further examination of SDO in minority groups would benefit from a more fine-grained examination of how different minority groups (with recent vs. distal immigration histories, and from different host nations) respond to their surroundings. At a macro level, such an approach fits with recent calls to recognize minority group differences, as well as similarities [29].

Returning to the present study, we believe our findings have implications for understanding broader social psychological phenomenon such as social identities, the social context, and relative deprivation. In particular, in support of past work from a social identity perspective [21] we see that the immediate social context is key to determining not only the strength of identity, but also its meaning (i.e., whether one’s group is perceived as high or lower power). Our work also suggests that personal SDO is inextricably linked to both group membership and the immediate environment. This is important, as SDO is sometimes conceptualized as a personality trait, whereas our work is more in line with an understanding of SDO that recognizes the role of group socialization [19]. Further work could extend this examination, by looking explicitly at the strength of minority group members’ social identity. For example, it is plausible that when minority group members are surrounded by outgroup members (as when the other-total ratio increases; [21]), their identification with their group increases. This might then prompt members of this group to recognize intergroup injustice, which in turn is associated with a reduction in support for the existing social hierarchy that ostensibly marginalizes them (i.e., SDO). Future research should therefore consider examining identification with the ingroup and perceptions of intergroup injustice as other potential mechanism through which outgroup density is associated with lower levels of SDO among minority group members. These additional mechanisms are leant support from the previous research which has found that ingroup salience and feeling relatively close to one’s ingroup (both features of ingroup identification; [30]) is negatively related to SDO among lower status group members [31]. Future research should pursue this additional mechanism as well as other mechanisms through which outgroup density is associated with lower levels of SDO among minority group members.

In this paper we made our argument using correlational data. Indeed, it would be very hard to test our argument experimentally, as we suggest that it is the day-to-day, lived experience of being surrounded by outgroup members that decreases minorities’ perceptions of their own group’s status, and consequently SDO. However, we acknowledge that there may be bidirectionality at play. It may be the case that minority group members who are high in SDO selectively move to neighborhoods in which they do not have to be reminded of their group’s relatively low status (e.g., neighborhoods in which they would not have to be exposed to outgroup members). This possibility, however, appears unlikely. Census-level analysis of residential mobility suggests that White householders become more likely to leave their neighborhood as the proportion of racial minority householders increases (the *White flight* hypothesis; [32]). In depth analysis of patterns of migration suggest that White avoidance of predominantly Black or mixed neighborhoods in the U.S. is a primary driver of housing segregation, rather than Black avoidance of White neighborhoods ([33]). Further, barriers exist for members of disadvantaged minority groups when it comes to freely choosing their residential neighborhood, so it is more likely that their surroundings affect their level of SDO. This might also be one reason why we found no correlation between the extent to which White Americans live and work surrounded by other White Americans in Study 3 –as advantaged group members they have more choice about where they live (as opposed to who they work with). As a point of interest, for majority group members (i.e., Whites), the lack of an association between outgroup density and SDO suggests that it is unlikely SDO is playing a key role in leaving neighborhoods populated with minority group members (i.e., White flight).

Another alternative explanation for our data is that extraneous variables help account for the relationship between outgroup density and SDO. It should be noted, though, that many extraneous variables (age, gender, level of education, and socio-economic status) were controlled for in the analysis. Moreover, we show that our data is not contaminated by participants misrepresenting the proportion of outgroup members in their immediate environment. Study 1 found that minority group members who lived in environments where there was an objectively high proportion of outgroup members reported lower SDO. This finding rules out the possibility that high SDO participants inflated the numerical dominance of their own group. Finally, it is difficult to identify who the participants are comparing themselves to in the perceived status measure. It is unknown whether participants are comparing themselves to White Americans or other minority groups (i.e., Hispanics, immigrants), both of which they may perceive as having more power than their group in their immediate environment.

### Conclusions

Broadly, we demonstrate that for minority group members, their immediate environment is associated with their endorsement of SDO. Minority group members who are not surrounded by other ingroup members are likely exposed to reminders of low ingroup status in their surrounding environment, which in turn predicts decreased SDO. This paper makes an important step forward in exploring SDO among racial minorities.

## Supporting information

S1 Dataset Dataset Study 2A. (SAV)

S2 Dataset Dataset Study 2B. (SAV)

S3 Dataset Dataset Study 3A. (SAV)

S4 Dataset Dataset Study 3B. (SAV)

S5 Dataset Dataset Study 4. (SAV)
